# Supplementary material for: A novel flexible near-infrared endoscopic device that enables real-time artificial intelligence fluorescence tissue characterization
Source: PLoS One. 2025 Mar 13;20(3):e0317771. doi: 10.1371/journal.pone.0317771 (PMC11906065; doi:10.1371/journal.pone.0317771)
Supplement: Attachment — Submitted filename: Response to reviewers.docx [file pone.0317771.s002.docx]

18^th^ December 2024.

Regarding the reviewer specific comments please find here point by point commentary:

**Comments to the Author** *1. Is the manuscript technically sound, and do the data support the conclusions? The manuscript must describe a technically sound piece of scientific research with data that supports the conclusions. Experiments must have been conducted rigorously, with appropriate controls, replication, and sample sizes. The conclusions must be drawn appropriately based on the data presented. Reviewer #1: Partly*

Author reply: We have carefully and explicitly delineated the construction via assembly of the device that is the subject of the report in such a way to allow others do so similarly and now supply a revised manuscript that includes the specific comments of the editorial office and indeed your own review. We agree this makes for a clearer, better structured report thank you! Please let us know if any further detail would be helpful to strengthen the report.

*2. Has the statistical analysis been performed appropriately and rigorously? Reviewer #1: N/A*

Author reply: We agree that statistical analysis is not a significant part of the meaning and message of the report, thank you.

*3. Have the authors made all data underlying the findings in their manuscript fully available? The* [*PLOS Data policy*](http://track.editorialmanager.com/CL0/http:%2F%2Fwww.plosone.org%2Fstatic%2Fpolicies.action%23sharing/1/010f0193b8a754c2-18adb94d-2676-4f15-8847-4776707042c1-000000/Qq0EKL60usE_GbBY3HCfA944xb7ddNrVRRJTRWp46LY=189) *requires authors to make all data underlying the findings described in their manuscript fully available without restriction, with rare exception (please refer to the Data Availability Statement in the manuscript PDF file). The data should be provided as part of the manuscript or its supporting information, or deposited to a public repository. For example, in addition to summary statistics, the data points behind means, medians and variance measures should be available. If there are restrictions on publicly sharing data—e.g. participant privacy or use of data from a third party—those must be specified. Reviewer #1: Yes*

Author reply: We agree!

*4. Is the manuscript presented in an intelligible fashion and written in standard English? PLOS ONE does not copyedit accepted manuscripts, so the language in submitted articles must be clear, correct, and unambiguous. Any typographical or grammatical errors should be corrected at revision, so please note any specific errors here. Reviewer #1: Yes*

Author reply: Thank you!

*5. Review Comments to the Author. Please use the space provided to explain your answers to the questions above. You may also include additional comments for the author, including concerns about dual publication, research ethics, or publication ethics. (Please upload your review as an attachment if it exceeds 20,000 characters) Reviewer #1: Dear Authors, I read you article presenting your new colonoscope-compatible flexible imaging probe for NIR visualization with its AI algorithm for ICG quantification. It raised my interest as well as some concerns over its structure.*

Author reply: Thank you for your careful review and comments, which we address now here below in order.

*I listed them as follows:- the structure of the article is not clear in my mind. It is a research article but it seems more of a leaflet presenting a new instrument. Actually it is not an ex-vivo study investigating the application of the instrument, nor it is a clinical article. Hence, what's the aim of the study?*

Author reply: The aim is to manifest a method that closes the current clinical gap in near infrared imaging articulated in the manuscript. That is that current NIR imagers aren’t flexible and flexible imagers don’t have NIR. We have revised the abstract and introduction to make this clearer. Thanks for the recommendation!

*- overall, the flow of the article should be increased. There are too many parenthesis, making it difficult to read.*

Author reply: we have revised the manuscript fully with this specific comment in mind and have, as you recommend, improve the flow for instance by making simpler, shorter sentences where appropriate. We agree this has greatly helped the flow of the paper making the message clearer and indeed the report shorter! Thank you!

*- Methods section should be deeply revised in its structure. Please describe the design of the study and insert subsections, more than you did (i.e., description of the instrument)*

Thank you, this has now been done. No problem to address further if you think could be improve further.

*- It seems like the clinical testing section of the results is halved or has a missing part*

This has been corrected, we agree it looked like a piece was missing but this has now been cleaned up.

*- Please discuss the potential clinical application of AI for the detection of ICG in different settings, still evaluating the bowel (i.e., perfusion, PMID: 33352315)*

This is now done with inclusion of your suggested reference and some others. Thank you!
